# EGFR DNA Methylation Correlates With EGFR Expression, Immune Cell Infiltration, and Overall Survival in Lung Adenocarcinoma

**DOI:** 10.3389/fonc.2021.691915

**Published:** 2021-08-10

**Authors:** Zhanyu Xu, Fanglu Qin, Liqiang Yuan, Jiangbo Wei, Yu Sun, Junqi Qin, Kun Deng, Tiaozhan Zheng, Shikang Li

**Affiliations:** ^1^Department of Thoracic and Cardiovascular Surgery, The First Affiliated Hospital of Guangxi Medical University, Nanning, China; ^2^School of Information and Management, Guangxi Medical University, Nanning, China

**Keywords:** lung adenocarcinoma, EGFR, DNA methylation, tumor biomarkers, tumor-infiltrating

## Abstract

**Background:**

The epidermal growth factor receptor (EGFR) is a primary target of molecular targeted therapy for lung adenocarcinoma (LUAD). The mechanisms that lead to epigenetic abnormalities of EGFR in LUAD are still unclear. The purpose of our study was to evaluate the abnormal methylation of EGFR CpG sites as potential biomarkers for LUAD.

**Methods:**

To assess the differentially methylation CpG sites of EGFR in LUAD, we used an integrative study of Illumina HumanMethylation450K and RNA-seq data from The Cancer Genome Atlas (TCGA). We evaluated and compared EGFR multiple-omics data to explore the role of CpG sites located in EGFR promoter regions and gene body regions and the association with transcripts, protein expression levels, mutations, and somatic copy number variation. We calculated the correlation coefficients between CpG sites of EGFR and immune infiltration fraction (by MCPcounter and ESTIMATE) and immune-related pathways in LUAD. Finally, we validated the differential methylation of clinically and prognostically relevant CpG sites using quantitative methylation-specific PCR (qMSP).

**Results:**

We found that the methylation level of many EGFR CpGs in the promoter region was negatively correlated with the transcription level, protein expression, and SCNV, while the methylation at the gene body region was positively correlated with these features. The methylation level of EGFR CpGs in the promoter region was positively correlated with the level of immune infiltration and IFN-γ signature, while the opposite was found for methylation of the gene body region. The qMSP results showed that cg02316066 had a high methylation level, while cg02166842 had a low methylation level in LUAD. There was a high degree of co-methylation between cg02316066 and cg03046247.

**Conclusion:**

Our data indicate that EGFR is an epigenetic regulator in LUAD acting through DNA methylation. Our research provides a theoretical basis for the further detection of EGFR DNA methylation as a predictive biomarker for LUAD survival and immunotherapy.

## Introduction

Lung cancer is the primary cause of cancer-related death worldwide (1). The most prominent pathological subtype of lung cancer is lung adenocarcinoma (LUAD), which accounts for about 45 percent of lung cancer cases (2). The five-years overall survival rate for patients with advanced lung cancer is less than 20% (3). Genetic analyses have revealed driver genes in LUAD and have changed the treatment paradigm (4). In Asia, epidermal growth factor receptor (EGFR) mutations account for 51.4% of advanced LUAD driver mutations, while it accounts for 15 to 22% of advanced LUAD driver mutations in non-Asian areas (5, 6). EGFR tyrosine kinase inhibitors (TKIs) are typically used to treat patients with EGFR-mutant LUAD (7, 8). The high heterogeneity of this type of cancer, on the other hand, restricts the survival advantage of patients undergoing EGFR-targeted treatment, indicating the need for more research into new prognosis-related molecular mechanisms. The molecular mechanism by which EGFR regulates LUAD through DNA methylation has yet to be completely elucidated.

EGFR is a receptor tyrosine kinase (TK) that dimerizes in response to ligand stimulation, resulting in the activation of intracellular TKs and autophosphorylation of multiple tyrosine residues, which triggers a sequence of downstream signaling cascades (9, 10). The Ras/MAPK and PI3K/PKB signaling pathways are two of the most studied EGFR pathways, both of which have a well-established role in tumor development, survival, and progression (11). The most studied epigenetic mechanism is DNA methylation, which is linked to cell division, immune regulation, and X chromosome inactivation (12). Methylation of gene promoter regions is often linked to transcriptional silencing, while methylation of gene bodies has the opposite effect. Epigenetic dysregulation has been linked to the early stages of oncogenic transformation in a variety of solid tumors, and it can be used as a biomarker for early detection, systemic sampling, and prognosis in a variety of human cancers (13). Indeed, Haijing Liu et al. (14) reported the potential link between EGFR alterations at the multi-omics levels and clinical prognosis by pan-cancer analysis, but the relationship between DNA methylation of EGFR in LUAD and immune infiltration has not been reported. For patients with LUAD who do not benefit from targeted therapy, the discovery of DNA methylation-related immune landscapes has important implications for the molecular mechanisms of immunotherapy.

Using the LUAD dataset from The Cancer Genome Atlas (TCGA), we performed a comprehensive multi-omics data assessment of EGFR-annotated CpGs. We investigated whether EGFR CpG methylation sites correlated with EGFR gene expression, protein levels and overall survival (OS) time. We further explored the relationship between somatic copy number variation (SCNV) and DNA methylation of EGFR, and the link between EGFR CpG methylation sites and LUAD immunological infiltration cells and immune-related pathways.

## Materials and Methods

### EGFR CpGs and TCGA Data Analysis

**Figure 1:**
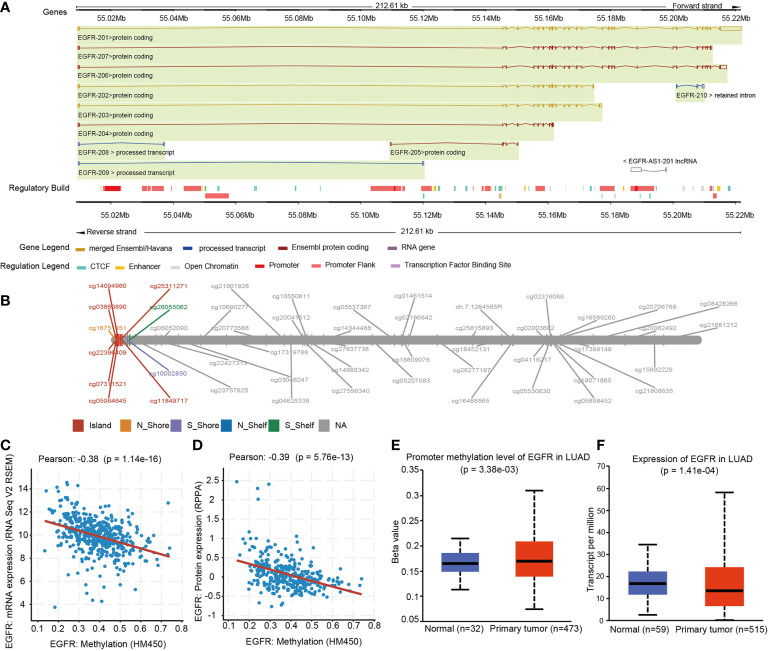
EGFR Genomic structure, CpG site landscape, methylation level. **(A)** Schematic representation of the EGFR gene structure within the human hg19 genome sequence. **(B)** Overview of 49 analyzed methylation sites of EGFR. **(C)** Correlation analysis between DNA methylation and mRNA expression of EGFR in the TCGA LUAD cohort. **(D)** Correlation analysis between DNA methylation and protein expression of EGFR in the TCGA LUAD cohort. **(E)** Promoter methylation levels of EGFR on normal and LUAD tissues. **(F)** EGFR mRNA expression in normal and LUAD groups.

The Illumina 450K TCGA dataset was used to extract methylation and expression data for all EGFR CpGs. We investigated 49 CpGs in the promoter and gene body regions of EGFR. **Figure 1** depicts the EGFR genome arrangement (**Figure 1A**) and the relative locations of all EGFR CpGs genomes (**Figure 1B**). Clinical information is integrated with data from the GDC data portal (https://portal.gdc.cancer.gov) (15). These data included 535 LUAD patients, as well as information on EGFR status and somatic copy number variation (SCNV). Methylation data came in the form of beta-values, while expression data came in the form of TPM (Transcripts Per kilobase Million)-normalized read counts. cBioProtal (http://www.cbioportal.org/study?id=luad_tcga#summary) was used to retrieve RPPA (Reverse phase protein array)-based protein expression data (16).

### Survival Analysis

For all available LUAD samples, Kaplan-Meier survival analysis curves (17) for the 49 EGFR CpG were plotted, with a P-value of 0.05 used as a statistical threshold, according to the group with high or low methylation. We did the above survival analysis curves in the EGFR wild-type group, EGFR mutation group and EGFR wild-type and PDL1 high expression group.

### Assessment of Immune Cells Infiltration, ESTIMATE Scores

We employed the R package ESTIMATE (18) to investigate the immune invasion of LUAD samples. After that, to obtain a more detailed picture of immune cell-types and other stromal cells infiltration, R package MCPcounter was used (19). MCPcounter utilizes the scoring data for individual tumor specimens (20). We calculated the Pearson correlation between the β value of the CpG site on EGFR and the score of immune infiltration.

### Sample Collection

20 paired LUAD and non-cancerous lung tissue samples were obtained from the First Affiliated Hospital of Guangxi Medical University from September 2019 to February 2020, and stored at -80°C. LUAD diagnosis was confirmed by two independent pathologists. This study was approved by the ethics committee of the First Affiliated Hospital of Guangxi Medical University.

### DNA Extraction, DNA Sodium Bisulfite Conversion, and Quantitative Methylation Specific PCR

Genomic DNA was isolated from individual specimens using a CTAB DNA extraction (21). Nanodrop 2000 spectrophotometer (Thermofisher, USA) was used to detect the concentration and purity of the DNA extraction, and nucleic acid gel electrophoresis was used to detect DNA integrity (22). Samples were then bisulfite-converted using the Epitect Fast DNA Bisulfite Kit (Qiagen; 59824), according to the manufacturer protocol. The purified products were quantitated using a Qubit ssDNA Assay kit (Thermo, Q10212). Primer Premier 6.0 software (Premier, Canada) was used to design the primer sequences to target CpG sites. Fully methylated genomic DNA after bisulfite treatment and normal genomic DNA (not transformed with bisulfite) were used as templates. Uncalibrated methylation levels, roughly equivalent to percent methylation, were calculated using cycle threshold (CT) values obtained from probes that specifically bind to methylated (CT_methylated_) and unmethylated (CT_unmethylated_) DNA, respectively (methylation [%] =100%/(1 + 2^CTmethylated–CTunmethylated^). The primers were utilized as shown in **Table 1**. QMSP was performed using an Applied Biosystems 7900HT Fast Real-Time PCR system (Waltham, Massachusetts, USA) with the following temperature profile: 5 minutes at 95°C, followed by 40 cycles of 15 seconds at 95°C, 30 seconds at 60°C, and 60 seconds at 60°C.

**Table 1:** Primer sequences used in this study.

Name	Sequence (5’-3’)	Types	Products (bp)
cg02316066-M	TGTGGGGTTACGGGTAAGTTTC	Forward Primer	170
cg02316066-M	TCTACCAATTATAAATCTAATATCACATAC	Reverse Primer	
cg02316066-U	TGTGGGGTTATGGGTAAGTTTT	Forward Primer	170
cg02316066-U	TCTACCAATTATAAATCTAATATCACATAC	Reverse Primer	
cg03046247-M	TGGAAATAGTATAAATTGGAGGTGA	Forward Primer	228
cg03046247-M	AACTACGCTATTTTAAAAACCACG	Reverse Primer	
cg03046247-U	TGGAAATAGTATAAATTGGAGGTGA	Forward Primer	228
cg03046247-U	AAAAACTACACTATTTTAAAAACCACA	Reverse Primer	
cg02166842-M	GAGTGAGTGGGTTTAGTTAAGTGAGT	Forward Primer	170
cg02166842-M	ACCCTCCTAAATATAATATTTACACG	Reverse Primer	
cg02166842-U	GAGTGAGTGGGTTTAGTTAAGTGAGT	Forward Primer	170
cg02166842-U	AACCCTCCTAAATATAATATTTACACA	Reverse Primer	

M, methylated; U, unmethylated.

### Statistical Analysis

Pearson correlation coefficients were used to assess correlations between EGFR mRNA expression, protein expression, immune score, and all individual beta values of EGFR in the TCGA dataset. Wilcoxon rank sum test with continuity correction was used to assess differential methylation. Results were deemed significant if p<0.05.

## Results

### Differential Methylation Analysis of EGFR and Its CpG Sites

We sought to explore whether variations in DNA methylation were linked to EGFR expression abnormalities. The cbioprotal official website analysis showed that EGFR hypermethylation is inversely correlated with mRNA (r^2^ = -0.38, *P* = 1.14e-16) (**Figure 1C**) and protein (r^2^ = -0.39, *P* = 5.76e-13) (**Figure 1D**) overexpression in LUAD of TCGA. We next analyzed the relationship between the methylation levels of the EGFR promoter and the clinicopathological parameters of LUAD patients by UALCAN. EGFR were significantly hypermethylated in LUAD tissues when compared with normal lung tissues (*P* = 3.38e-3) (**Figure 1E**), and the mRNA levels of EGFR in LUAD were remarkably lower than those in normal lung tissues (*P* = 1.41e-4) (**Figure 1F**).

In the same cohort of LUAD patients from the TCGA database we collected methylation data from the Infinium HumanMethylation450 BeadChip for 49 CpG sites of EGFR (**Table 2** and **Supplemental Table 1**). Six CpG sites were located in promoter regions (cg16751451, cg07311521, cg03860890, cg22396409, cg05064645, cg14094960) and 43 were in the gene body or in the 3’ UTR regions. There were 34 of 49 CpG sites that were differentially methylated between LUAD tissues and control groups (*P* < 0.05). Five CpG sites in promoter regions and 17 CpG sites in the gene body had a significantly higher percentage of methylation in LUAD when compared to normal lung tissues. Meanwhile, cg16751451 in promoter regions and 11 CpG sites in the gene body or 3’ UTR regions had a significantly lower percentage of methylation in LUAD compared to normal lung tissues.

**Table 2:** Differential methylation levels of EGFR CpG sites among different subgroups.

CpG site	Position	Mean methylation level		p value	Mean methylation level		p value	Mean methylation level					p value
		normal	LUAD		EGFR-mutation	EGFR-wild		-2	-1	0	1	2	
**cg03860890**	TSS1500	0.13	0.16	**9.80E-04**	0.14	0.16	**3.40E-04**	0.18	0.18	0.16	0.15	0.14	**2.30E-02**
**cg05064645**	5’UTR;1stExon	0.05	0.08	**4.00E-04**	0.06	0.08	**1.10E-02**	0.20	0.09	0.09	0.07	0.06	**3.90E-07**
**cg07311521**	TSS1500	0.03	0.05	**3.90E-03**	0.04	0.05	**6.60E-03**	0.04	0.05	0.05	0.05	0.06	8.10E-01
**cg14094960**	5’UTR;1stExon	0.08	0.11	**4.70E-05**	0.10	0.11	1.70E-01	0.24	0.12	0.12	0.10	0.09	**1.50E-06**
**cg16751451**	TSS1500	0.38	0.36	**1.50E-02**	0.30	0.36	**1.00E-04**	0.42	0.39	0.36	0.35	0.33	2.50E-01
**cg22396409**	TSS1500	0.13	0.16	**1.20E-02**	0.15	0.16	**3.80E-02**	0.15	0.17	0.16	0.16	0.16	7.50E-01
cg01461514	Body	0.59	0.39	**2.20E-15**	0.32	0.40	**7.20E-07**	0.49	0.47	0.40	0.39	0.26	**3.70E-10**
cg02003682	Body	0.81	0.76	**1.00E-04**	0.77	0.75	**3.60E-02**	0.72	0.71	0.75	0.77	0.74	**4.00E-03**
cg02166842	Body	0.60	0.53	**2.60E-03**	0.42	0.54	**2.40E-10**	0.59	0.58	0.53	0.53	0.42	**7.30E-04**
cg02316066	Body	0.63	0.67	**3.50E-03**	0.69	0.66	**6.60E-03**	0.58	0.65	0.65	0.68	0.71	**1.40E-04**
cg03046247	Body	0.73	0.70	1.30E-01	0.71	0.70	1.30E-01	0.62	0.70	0.69	0.71	0.71	**2.50E-02**
cg04116217	Body	0.79	0.76	**3.20E-02**	0.77	0.76	8.30E-02	0.67	0.72	0.75	0.77	0.74	**2.50E-02**
cg04625338	Body	0.31	0.29	**1.30E-02**	0.28	0.29	4.80E-01	0.25	0.30	0.28	0.31	0.26	3.00E-01
cg05207583	Body	0.74	0.76	**1.60E-04**	0.72	0.77	**1.20E-04**	0.66	0.77	0.77	0.77	0.72	**2.70E-03**
cg05530630	Body	0.79	0.80	**2.90E-02**	0.80	0.80	**4.10E-02**	0.72	0.80	0.79	0.81	0.80	**1.60E-02**
cg05537387	Body	0.81	0.80	8.10E-01	0.76	0.80	**1.10E-02**	0.72	0.79	0.79	0.81	0.76	**2.00E-02**
cg05898452	Body	0.59	0.61	1.20E-01	0.63	0.60	**1.10E-02**	0.46	0.58	0.59	0.62	0.64	**2.40E-04**
cg06052090	Body	0.67	0.74	**5.80E-07**	0.68	0.75	**1.30E-03**	0.68	0.74	0.72	0.75	0.74	6.10E-02
cg10002850	Body	0.70	0.80	**1.60E-06**	0.69	0.81	**1.30E-05**	0.83	0.82	0.81	0.80	0.68	**4.70E-03**
cg10550611	Body	0.76	0.72	2.80E-01	0.70	0.72	2.30E-01	0.73	0.71	0.72	0.72	0.70	9.10E-01
cg10690277	Body	0.86	0.85	1.20E-01	0.84	0.85	2.60E-01	0.79	0.85	0.85	0.85	0.82	**7.40E-03**
cg11849717	Body	0.12	0.12	4.40E-01	0.11	0.12	**9.10E-03**	0.16	0.13	0.13	0.11	0.11	**2.00E-03**
cg14344486	Body	0.70	0.70	8.80E-02	0.60	0.72	**1.50E-10**	0.75	0.73	0.72	0.70	0.60	**1.80E-04**
cg14688342	Body	0.48	0.43	**3.30E-04**	0.42	0.43	3.30E-01	0.42	0.42	0.43	0.43	0.42	9.30E-01
cg15692229	Body	0.73	0.86	**2.20E-16**	0.85	0.86	7.40E-01	0.86	0.86	0.85	0.86	0.84	1.30E-01
cg16488565	Body	0.54	0.62	**4.90E-10**	0.63	0.62	**3.60E-02**	0.51	0.60	0.61	0.63	0.61	**5.70E-04**
cg16589260	Body	0.76	0.79	**1.70E-03**	0.79	0.79	5.40E-01	0.74	0.81	0.79	0.79	0.78	8.40E-02
cg17319788	Body	0.85	0.81	3.60E-01	0.80	0.82	3.30E-01	0.79	0.81	0.83	0.82	0.68	**2.70E-10**
cg17389149	Body	0.86	0.85	8.70E-01	0.86	0.85	**2.30E-02**	0.83	0.82	0.84	0.86	0.86	**2.40E-02**
cg18071865	Body	0.58	0.61	**3.30E-03**	0.63	0.61	9.40E-02	0.51	0.59	0.60	0.63	0.64	**8.80E-04**
cg18452131	Body	0.83	0.79	**2.70E-04**	0.79	0.79	5.50E-01	0.71	0.78	0.78	0.80	0.76	**2.10E-02**
cg18809076	Body	0.79	0.79	5.10E-02	0.71	0.80	**1.00E-09**	0.78	0.82	0.80	0.78	0.65	**3.70E-10**
cg20041612	Body	0.70	0.64	2.60E-01	0.60	0.65	**7.80E-03**	0.55	0.62	0.66	0.64	0.60	1.50E-01
cg20062492	Body	0.79	0.73	**1.30E-06**	0.73	0.72	1.50E-01	0.64	0.72	0.72	0.74	0.72	**2.30E-02**
cg20706768	Body	0.75	0.78	**5.20E-04**	0.78	0.78	9.20E-01	0.67	0.78	0.77	0.79	0.81	**1.20E-04**
cg20773588	Body	0.74	0.78	**5.60E-04**	0.79	0.78	5.10E-02	0.61	0.77	0.77	0.80	0.73	**9.00E-04**
cg21681212	Body	0.60	0.74	**4.90E-14**	0.72	0.74	7.70E-02	0.68	0.73	0.73	0.74	0.71	8.70E-02
cg21808635	Body	0.77	0.83	**6.00E-06**	0.84	0.83	3.70E-01	0.71	0.83	0.82	0.83	0.86	**2.70E-04**
cg21901928	Body	0.73	0.75	**5.60E-03**	0.74	0.75	3.70E-01	0.66	0.72	0.74	0.76	0.74	**3.80E-02**
cg22427313	Body	0.68	0.69	1.00E-01	0.68	0.69	6.00E-01	0.65	0.64	0.66	0.71	0.69	**9.90E-04**
cg23757825	Body	0.88	0.84	1.70E-01	0.82	0.85	**5.80E-03**	0.76	0.81	0.85	0.85	0.80	**7.80E-03**
cg25311271	Body	0.09	0.10	**3.00E-03**	0.10	0.11	6.10E-02	0.14	0.11	0.11	0.10	0.09	**1.30E-03**
cg25815893	Body	0.82	0.80	2.00E-01	0.81	0.80	**4.80E-02**	0.73	0.78	0.80	0.81	0.77	**1.80E-02**
cg26055062	Body	0.68	0.76	**1.70E-08**	0.68	0.77	**1.70E-05**	0.73	0.78	0.76	0.76	0.72	4.90E-01
cg26277197	Body	0.41	0.39	1.10E-01	0.31	0.40	**6.00E-09**	0.51	0.43	0.41	0.37	0.28	**3.30E-08**
cg27598340	Body	0.95	0.85	**4.20E-03**	0.88	0.84	2.00E-01	0.65	0.77	0.83	0.87	0.84	**2.30E-02**
cg27637738	Body	0.48	0.53	**2.70E-02**	0.42	0.54	**1.70E-07**	0.51	0.60	0.49	0.55	0.48	**4.30E-05**
ch.7.1264585R	Body	0.12	0.11	**2.50E-02**	0.11	0.11	7.70E-01	0.10	0.11	0.11	0.11	0.10	8.20E-01
cg08428266	3’UTR	0.82	0.78	**1.30E-03**	0.78	0.77	1.10E-01	0.67	0.77	0.76	0.79	0.78	**3.20E-03**

(Somatic copy number variation type: -2, shallow deletion; -1, diploid; 0, normal; 1, gain; 2, amplification). CpG sites in bold values indicate located in the EGFR promoter region. The p-values in bold values indicate statistical differences (p < 0.05).

LUAD with EGFR mutations is a subtype of LUAD with a particular molecular mechanism and selective treatment (23). We analyzed EGFR methylation changes of LUAD in the EGFR mutations group and EGFR wild-type (non-mutated) group. We found that in the EGFR mutation group, EGFR showed a significant hypomethylation state. Interestingly, when performing differential methylation analysis of CpG sites we found that 19 of 26 CpGs were significantly hypomethylated while 7 CpGs were significantly hypermethylated in the EGFR mutation group compared to the EGFR wild-type group.

We further explored the relationship between SCNV and DNA methylation of EGFR, and found that there was a negative correlation between SCNV and DNA methylation for some CpGs (cg05064645, cg14094960, cg25311271, cg11849717, cg03860890), while for the majority (26/31 CpGs) there was a positive correlation.

### OS-Related CpG Sites

**Figure 2:**
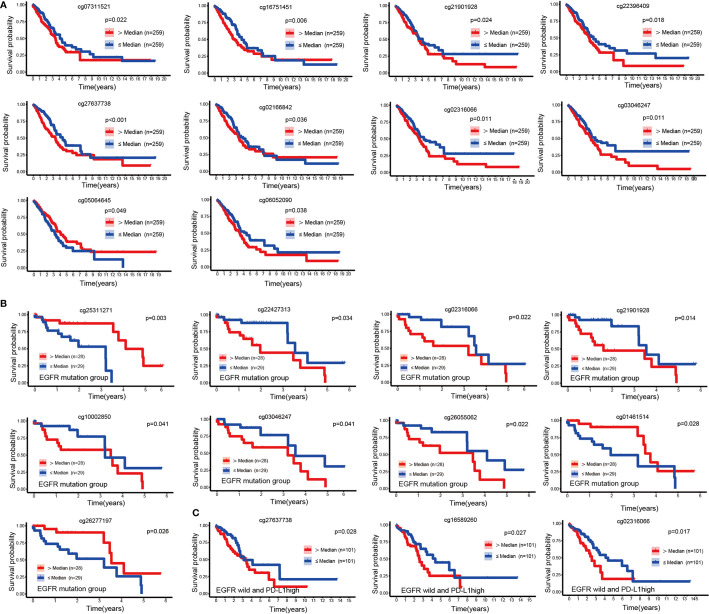
KM curves of EGFR CpG sites. **(A)** Kaplan-Meier analysis regarding overall survival in patients with LUAD stratified according to EGFR methylation CpG sites. **(B**, **C)** Kaplan-Meier analysis of the overall survival rate of LUAD patients based on the EGFR mutation group and the EGFR wild-type PDL1 high expression group.

To explore the prognostic value of 49 CpGs of EGFR in LUAD, we constructed survival curves to evaluate the association between CpGs and OS with the Kaplan-Meier method. A total of 10 CpG sites were significantly associated with the OS of LUAD patients (**Figure 2A**). Except for cg05064645, the hypermethylation of cg27637738, cg16751451, cg02316066, cg22396409, cg03046247, cg02166842, cg21901928, cg07311521 and cg06052090 CpG sites revealed poor prognosis of LUAD patients (p<0.05).

We next performed a survival analysis in the EGFR-mutant and EGFR-wild subsets separately. In the EGFR mutation group, the hypermethylation of cg01461514, cg26277197 and cg25311271 was associated with a good prognosis of LUAD (p<0.05), while the hypermethylation of cg21901928, cg22427313, cg02316066, cg26055062, cg10002850 and cg03046247 was associated with a poor prognosis (p<0.05) (**Figure 2B**). In patients with wild type EGFR, high levels of PDL1 expression affected the prognosis of immunotherapy (24). In the EGFR wild-type group, we divided LUAD patients into three equal parts according to the mRNA expression level of PDL1, and the group with the highest expression was identified as the EGFR wild-type and high PDL1 expression group. We found that the hypermethylation of cg02316066, cg16589260, and cg27637738 was associated with a poor prognosis in LUAD patients without EGFR mutations but with high PDL1 expression (p<0.05) (**Figure 2C**).

**Figure 3:**
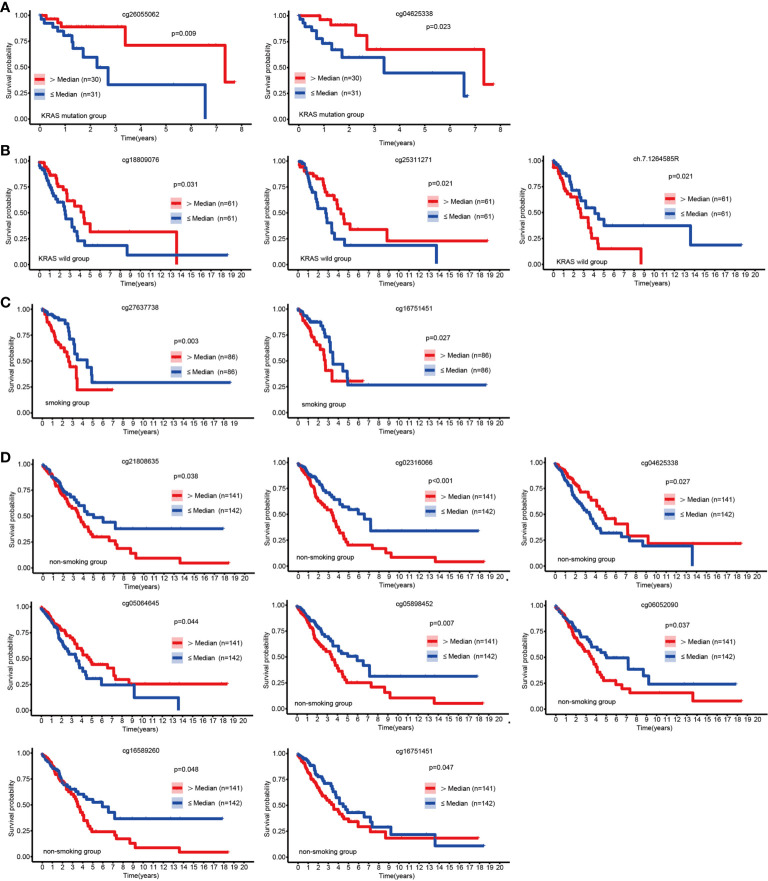
KM curves of EGFR CpG sites. Kaplan-Meier analysis of the overall survival rate of LUAD patients based on the KRAS mutation group **(A)**, KRAS wild-type group **(B)**, smoking group **(C)** and the non-smoking group **(D)**.

Apart from EGFR mutations, KRAS mutations were the most common mutations in LUAD (25), and we discovered that hypermethylation of cg26055062 and cg04625338 predicted good prognosis in LUAD patients with KRSA mutations (**Figure 3A**). Hypermethylation of cg18809076 and cg25311271 in the KRAS wild-type group predicted good prognosis, but ch.7.1264585R had the opposite effect (**Figure 3B**).

Patients with LUAD might have a variety of molecular features depending on their smoking history (26). We explored the relationship between EGFR methylation and prognosis in patients with LUAD in the smoking and non-smoking groups. Hypermethylation of cg16751451 and cg27637738 suggested a poor prognosis for LUAD in the smoking group (**Figure 3C**). In the nonsmoking group, hypermethylation of cg04625338 and cg05064645 indicated a favorable outcome, but cg02316066, cg05898452, cg06052090, cg21808635, cg16751451, and cg16589260 had the opposite effect (**Figure 3D**).

### EGFR Expression Is Correlated With DNA Methylation

**Figure 4:**
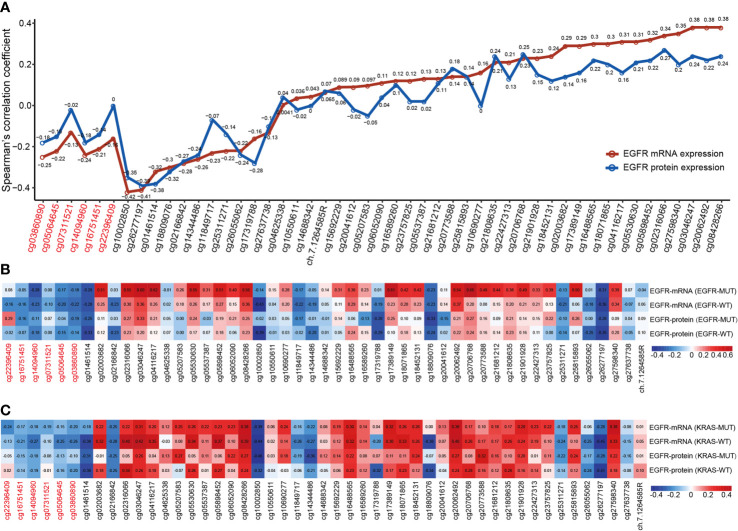
The correlation of EGFR CpG sites methylation and mRNA and protein expression of EGFR in LUAD. **(A)** Correlation analysis between DNA methylation levels of 49 EGFR CpG sites and mRNA and protein expression of EGFR in the TCGA LUAD cohort. **(B, C)** Correlation analysis between DNA methylation levels of 49 EGFR CpG sites and mRNA and protein expression of EGFR in EGFR and KRAS status groups of TCGA LUAD cohort. CpG sites labeled in red are located in the EGFR promoter region and in black in the gene body region.

Individual CpG methylation was studied in relation to EGFR mRNA and protein expression (**Figure 4A**). Of the 49 CpG sites examined in LUAD tissue, 44 had a strong association with EGFR mRNA expression. The methylation levels of all CpG sites in promoter regions were shown to be inversely correlated to EGFR mRNA levels. The methylation level of CpG sites in 27 of 43 CpGs within the gene body region was positively correlated with the mRNA level of EGFR, and 11/43 showed a significant negative correlation. Cg03046247, cg08428266 and cg20062492 hypermethylation was significantly related to the high expression of EGFR mRNA, and cg10002850 hypomethylation was significantly related to the high expression of EGFR mRNA. Then we investigated the connection between CpG methylation and EGFR protein expression, and the findings matched the EGFR mRNA association described previously. Cg02316066 hypermethylation and cg01461514 hypomethylation were significantly related to the high expression of EGFR protein.

Next, we explored the correlation between the mRNA and protein expression levels of EGFR and EGFR CpG sites in EGFR and KRAS mutation status or not (**Figures 4B, C**). Overall, similar to the above results, the β values of CpG sites located in the EGFR promoter region were negatively correlated with the mRNA and protein levels of EGFR in both the EGFR mutant and KRAS mutant groups, while the CpG sites of the gene body showed the opposite effect. Interestingly, we found a higher correlation, both positive and negative, in the EGFR mutant group compared to the EGFR wild-type group. In contrast, this correlation was lower in the KRAS mutation group than in the KRAS wild-type group.

### EGFR Methylation and Expression Are Associated With Immune Cells Infiltration

**Figure 5:**
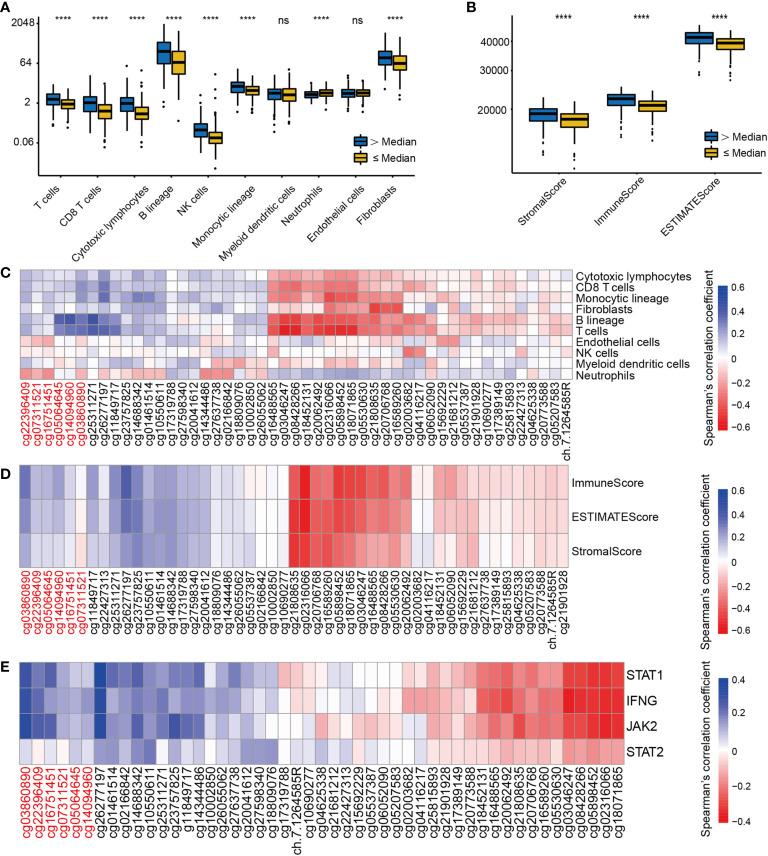
The correlation of EGFR CpG sites methylation and immune cells infiltrates and IFN-γ signature in LUAD. **(A)** The landscape of immune infiltration in LUAD according to the median EGFR methylation level by MCPcounter. **(B)** ESTIMATE scores in LUAD according to the median EGFR methylation level. **(C–E)**Correlation analysis between DNA methylation levels of 49 EGFR CpG sites and immune infiltration, ESTIMATE scores, IFN-γ signature in the TCGA LUAD cohort. CpG sites labeled in red are located in the EGFR promoter region and in black in the gene body region. ****P < 0.0001, ns, not significant.

Immune cells from a variety of species are known to infiltrate the tumor microenvironment (27). We explored the association between EGFR methylation levels and the infiltration levels of 8 immune cells and 2 stromal cells of MCPcounter. Based on the median EGFR integrated methylation level, we divided the LUAD samples into hypermethylated and hypomethylated EGFR groups. The results showed that the EGFR hypermethylation group was associated with increased infiltration of T cells, CD8 T cells, cytotoxic lymphocytes, B lineage, NK cells, monocytic lineage, and fibroblasts (**Figure 5A**). Moreover, the immune score, stromal score and estimate score of ESTIMATE were higher in the EGFR hypermethylated group than those of the hypomethylated group (**Figure 5B**). We found a positive association between EGFR promoter hypermethylation and the infiltration of T cells, CD8 T cells, cytotoxic lymphocytes, B lineage, NK cells, monocytic lineage, endothelial cells and fibroblasts, while EGFR body hypermethylation had lower infiltration of the above immune cells (**Figure 5C**). To further examine this scenario, we found that EGFR promoter hypermethylation had higher immune scores (p<0.001), a marker of total immune infiltration (**Figure 5D**). The association between EGFR methylation and the IFN-signature was also investigated. Increased mRNAs of the main IFN-signature genes (IFNG, STAT1, STAT2, JAK2) were found to be linked to extensive promoter hypermethylation and gene body hypomethylation (**Figure 5E**).

### Validation of CpG Site Methylation by qMSP in External Cohorts

**Figure 6:**
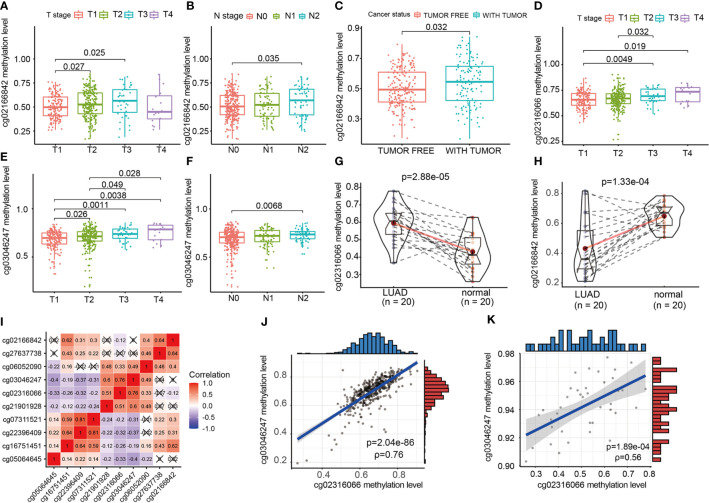
The relationship of EGFR CpG sites DNA methylation levels and clinicopathologic parameters. **(A)** Association of cg02166842 DNA methylation levels with T stage. **(B)** Association of cg02166842 DNA methylation levels with N stage. **(C)** Association of cg02166842 DNA methylation levels with cancer status. **(D)** Association of cg02316066 DNA methylation levels with T stage. **(E)** Association of cg03046247 DNA methylation levels with T stage. **(F)** Association of cg03046247 DNA methylation levels with N stage. **(G)** DNA methylation levels of cg02316066 in normal and LUAD samples. **(H)** DNA methylation levels of cg02166842 in normal and LUAD samples. **(I)** Correlation heat map of DNA methylation levels at 10 OS-associated CpG sites. **(J)** Association between the DNA methylation of cg02316066 and cg03046247 in the TCGA LUAD cohort. **(K)** Association between the DNA methylation of cg02316066 and cg03046247 in external validation cohort.

We evaluated the relationship between CpG sites and clinical information, such as *T* staging, *N* staging and cancer states. As shown in **Figure 6**, the increase in the methylation β value of cg02166842 was significantly correlated with the T stage (T1 vs T2, T1 vs T3) (p=0.027, p=0.025 respectively) (**Figure 6A**), N stage (N0 vs N2) (**Figure 6B**), (p=0.035) and with tumor status (p=0.032) (**Figure 6C**). The methylation β value of cg02316066 was positively correlated with the T stage (T1 vs T3, T1 vs T4, T2 vs T3) (p=0.0049, p=0.019, p=0.032 respectively) (**Figure 6D**), while the methylation β value of cg03046247 was positively correlated with the T stage (T1 vs T2, T1 vs T3, T1 vs T4, T2 vs T3,T2 vs T4) (p=0.026, p=0.0011, p=0.0038, p=0.049, p=0.028 respectively) (**Figure 6E**) and N stage (N0 vs N2) (p=0.0068) (**Figure 6F**). To determine whether there was a difference in the methylation levels of cg02316066, cg03046247 and cg02166842 between LUAD tissue and adjacent tissues, we collected 20 pairs of tissue specimens. Consistent with the TCGA database results, cg02316066 showed hypermethylation levels and cg02166842 showed hypomethylation in LUAD (**Figures 6G, H**). Ten OS-related CpG sites showed high correlation coefficients towards each other indicating a high degree of co-methylation (**Figure 6I**). We noted that cg02316066 and cg03046247 were strongly associated with multiple types of clinical profiles and LUAD prognosis, and there was also a high degree of co-methylation between cg02316066 and cg03046247. In the TCGA cohort, the cg02316066 and cg03046247 Pearson correlation coefficient was 0.76 (p<0.001) (**Figure 6J**) and, consistent with this, in our validation cohort the correlation coefficient was 0.56 (p<0.001) (**Figure 6K**).

## Discussion

So far, EGFR is a consensual factor that promotes cancer progression and the development of EGFR-TKIs has dramatically changed the therapeutic landscape for patients with non-small cell lung cancer (28). However, the rapid occurrence of clinical drug resistance hinders patient survival (8). TKIs or monoclonal antibodies targeting EGFR can block the infiltration of immunosuppressive cells and improve the antitumor response in NSCLCs, indicating that combining EGFR-targeted therapy with immune checkpoint inhibitors is a viable alternative for combination immunotherapy (29).

Epigenetic variations are being gradually investigated, and they are now changing the idea that malignant lesions depend entirely on genetic expressions to develop (30). DNA methylation, which is largely responsible for gene silencing and chromatin formation, is by far the most studied epigenetic regulatory mechanism (31). Methyl groups are covalently bound to cytosine during DNA methylation to generate 5-methylcytosine (5mC) (32). Furthermore, DNA methylation is chemically stable, can be tested separately, and has strong biomarker potential (33). Methylation quantitative measurement of small samples (microanatomical cells, biopsies) is often performed in the clinic. These are the advantages of using methylation as a biomarker (34, 35).

To establish the various levels of methylation sites of EGFR in LUAD, we collected RNA-seq results and Illumina HumanMethylation450K from TCGA. We meticulously investigated DNA methylation at the EGFR 49 CpG sites. We correlated EGFR methylation with transcription and protein expression in LUAD tissues. Our results indicate that DNA methylation in the promoter and gene body regions resulted in strong epigenetic regulation of EGFR. In the LUAD TCGA cohort, hypomethylation of the promoter region was negatively associated with increased mRNA and protein expression, while hypomethylation in the gene body was nearly always positively correlated with EGFR expression. This strong epigenetic regulation of EGFR is present not only in the different mutational states of EGFR but also in the KRAS mutant and wild-type groups, and interestingly, mutations in EGFR enhance this epigenetic regulation of EGFR, while mutations in KRAS attenuate this property. Some studies have revealed that EGFR and KRAS mutations are mutually exclusive in lung adenocarcinoma (36), the mechanisms of which need to be investigated in more depth. These methylation defects can eventually affect the clinical characteristics and prognosis of LUAD patients.

Mutations in the Furin-like and Pkinase-Tyr domains were shown to be predictive indicators of effective TKI therapy for NSCLC (37–39), with slightly longer survival as compared to standard combination chemotherapy (40, 41). Different EGFR mutations have various benefits, and those that inhibit EGFR kinase activity can benefit from EGFR-targeted therapy (42, 43). In LUAD patients with an EGFR mutant phenotype, we found that most (19/26) CpG sites were hypomethylated and six of these were predictors for a good prognosis. There was a negative correlation between SCNV and DNA methylation at sites in CpG islands, and conversely, a positive correlation between sites in the CpG ocean of EGFR (26/31).

T cells, CD8 T cells, cytotoxic lymphocytes, B lineage, NK cells, monocytic lineage, and fibroblasts were found to be infiltrated more frequently in tissues that presented EGFR hypermethylation. Also, the immune score, stromal score and estimate score were higher in the EGFR hypermethylated group than those in the hypermethylated group. The non-inflammatory tumor microenvironment (TME) in EGFR-mutated NSCLCs is abundant in Treg cells and macrophages, with the latter releasing chemokines that attract more Treg cells in the inflammatory TME (44). EGFR-TKI therapy facilitates CD8+ T cell recruitment and prevents Treg cell infiltration in the TME in EGFR-mutated tumors *in vivo* (45). Therefore, we evaluated the relationship between EGFR methylation and an IFN-γ signature. Wide-spread promoter hypermethylation and body hypomethylation were strongly associated with increased IFN-γ signature. *In vitro* studies have shown that blocking EGFR with antibodies or kinase inhibitors facilitate the secretion of chemokines (CCL2, CCL5, and CXCL10) in HNSCC cells and keratinocytes when IFN and tumor necrosis factor (TNF) are stimulated (46).

In summary, our research shows that EGFR participates in the epigenetic regulation of LUAD through DNA methylation. DNA methylation of EGFR shows unique clinical characteristics and immunogenicity. Our research provides a theoretical basis for further assessment of EGFR DNA methylation, which can be used as a biomarker to predict the prognosis and immune mechanisms of LUAD.

## Data Availability Statement

The original contributions presented in the study are included in the article/**Supplementary Material**. Further inquiries can be directed to the corresponding author.

## Ethics Statement

The studies involving human participants were reviewed and approved by The Ethics Committee of the First Affiliated Hospital of Guangxi Medical University. Written informed consent for participation was not required for this study in accordance with the national legislation and the institutional requirements.

## Author Contributions

ZX and FQ conceived and designed the experiments, performed the qMSP experiments, prepared figures and/or tables and approved the final draft. LY, KD, and TZ performed bioinformatics analysis, analyzed the data, authored or reviewed drafts of the paper and approved the final draft. YS and JQ prepared figures and/or tables and approved the final draft. SL conceived and designed the experiments, approved the final draft. All authors contributed to the article and approved the submitted version.

## Funding

This study was supported by the National Natural Science Foundation of China (no. NSFC81660488) and the Guangxi Natural Science Foundation under grant no. 2017GXNSFAA198123.

## Conflict of Interest

The authors declare that the research was conducted in the absence of any commercial or financial relationships that could be construed as a potential conflict of interest.

## Publisher’s Note

All claims expressed in this article are solely those of the authors and do not necessarily represent those of their affiliated organizations, or those of the publisher, the editors and the reviewers. Any product that may be evaluated in this article, or claim that may be made by its manufacturer, is not guaranteed or endorsed by the publisher.

## Supplementary Material

Supplementary Table 1 Annotation information of 49 CpG sites of EGFR.
