# Supplementary material for: EGFR DNA Methylation Correlates With EGFR Expression, Immune Cell Infiltration, and Overall Survival in Lung Adenocarcinoma
Source: Front Oncol. 2021 Aug 10;11:691915. doi: 10.3389/fonc.2021.691915 (PMC8383738; doi:10.3389/fonc.2021.691915)
Supplement: Supplementary Table 1 — Annotation information of 49 CpG sites of EGFR. [file Table_1.docx]

**Supplementary Table 1. Annotation information of 49 CpG sites of EGFR**

| probeID | CpG_chrm | CpG_beg | CpG_end | probe_strand | Position |
| --- | --- | --- | --- | --- | --- |
| cg16751451 | chr7 | 55018397 | 55018399 | - | TSS1500 |
| cg22396409 | chr7 | 55018452 | 55018454 | + | TSS1500 |
| cg07311521 | chr7 | 55018470 | 55018472 | - | TSS1500 |
| cg03860890 | chr7 | 55018594 | 55018596 | - | TSS1500 |
| cg05064645 | chr7 | 55019174 | 55019176 | - | 5'UTR;1stExon |
| cg14094960 | chr7 | 55019196 | 55019198 | - | 5'UTR;1stExon |
| cg25311271 | chr7 | 55019825 | 55019827 | - | Body |
| cg11849717 | chr7 | 55020410 | 55020412 | - | Body |
| cg10002850 | chr7 | 55022076 | 55022078 | + | Body |
| cg26055062 | chr7 | 55022822 | 55022824 | - | Body |
| cg23757825 | chr7 | 55024577 | 55024579 | - | Body |
| cg22427313 | chr7 | 55031202 | 55031204 | - | Body |
| cg06052090 | chr7 | 55032914 | 55032916 | - | Body |
| cg03046247 | chr7 | 55038356 | 55038358 | - | Body |
| cg10690277 | chr7 | 55045505 | 55045507 | - | Body |
| cg17319788 | chr7 | 55053707 | 55053709 | + | Body |
| cg20773588 | chr7 | 55066407 | 55066409 | + | Body |
| cg21901928 | chr7 | 55072153 | 55072155 | + | Body |
| cg04625338 | chr7 | 55074277 | 55074279 | + | Body |
| cg27598340 | chr7 | 55078033 | 55078035 | - | Body |
| cg20041612 | chr7 | 55078679 | 55078681 | + | Body |
| cg14688342 | chr7 | 55079380 | 55079382 | - | Body |
| cg10550611 | chr7 | 55079464 | 55079466 | + | Body |
| cg27637738 | chr7 | 55080950 | 55080952 | + | Body |
| cg14344486 | chr7 | 55084704 | 55084706 | - | Body |
| cg05207583 | chr7 | 55100150 | 55100152 | + | Body |
| cg05537387 | chr7 | 55107305 | 55107307 | + | Body |
| cg01461514 | chr7 | 55109488 | 55109490 | + | Body |
| cg18809076 | chr7 | 55109929 | 55109931 | + | Body |
| cg02166842 | chr7 | 55111777 | 55111779 | - | Body |
| cg26277197 | chr7 | 55120938 | 55120940 | - | Body |
| cg25815893 | chr7 | 55132236 | 55132238 | + | Body |
| cg18452131 | chr7 | 55141519 | 55141521 | - | Body |
| ch.7.1264585R | chr7 | 55144946 | 55144947 | + | Body |
| cg16488565 | chr7 | 55145344 | 55145346 | + | Body |
| cg02003682 | chr7 | 55155851 | 55155853 | + | Body |
| cg04116217 | chr7 | 55155970 | 55155972 | - | Body |
| cg05530630 | chr7 | 55157049 | 55157051 | + | Body |
| cg05898452 | chr7 | 55157169 | 55157171 | + | Body |
| cg18071865 | chr7 | 55157201 | 55157203 | + | Body |
| cg02316066 | chr7 | 55157216 | 55157218 | + | Body |
| cg16589260 | chr7 | 55157388 | 55157390 | + | Body |
| cg21808635 | chr7 | 55157439 | 55157441 | + | Body |
| cg20706768 | chr7 | 55157489 | 55157491 | - | Body |
| cg17389149 | chr7 | 55160190 | 55160192 | + | Body |
| cg20062492 | chr7 | 55180878 | 55180880 | + | Body |
| cg15692229 | chr7 | 55190252 | 55190254 | + | Body |
| cg21681212 | chr7 | 55193219 | 55193221 | - | Body |
| cg08428266 | chr7 | 55170718 | 55170720 | - | 3'UTR |
